# Prevalence of Depression in Patients With Hypertension: A Systematic Review and Meta-Analysis: Erratum

**DOI:** 10.1097/MD.0000000000011059

**Published:** 2018-06-01

In the article, “Prevalence of Depression in Patients With Hypertension: A Systematic Review and Meta-Analysis”,^[1]^ which appeared in Volume 94, Issue 31 of *Medicine*, the link to the Supplementary Digital Content was not included. That link should be ht∗tp://links.lww.com/MD/C281.
